# EODA: A three-stage efficient outlier detection approach using Boruta-RF feature selection and enhanced KNN-based clustering algorithm

**DOI:** 10.1371/journal.pone.0322738

**Published:** 2025-05-30

**Authors:** Sunil Kumar, Sudeep Varshney, Usha Jain, Prashant Johri, Abdulaziz S. Almazyad, Ali Wagdy Mohamed, Mehdi Hosseinzadeh, Mohammad Shokouhifar

**Affiliations:** 1 School of Computing Science and Engineering, Galgotias University, Greater Noida, Uttar Pradesh, India; 2 Department of Computer Science & Engineering, School of Engineering & Technology, Sharda University, Greater Noida, India; 3 Department of Computer Science & Engineering, Manipal University Jaipur, Jaipur, India; 4 Department of Computer Engineering, College of Computer and Information Sciences, King Saud University, Riyadh, Saudi Arabia; 5 Operations Research Department, Faculty of Graduate Studies for Statistical Research, Cairo University, Giza, Egypt; 6 Applied Science Research Center, Applied Science Private University, Amman, Jordan; 7 Centre for Research Impact & Outcome, Chitkara University Institute of Engineering and Technology, Chitkara University, Rajpura, Punjab, India; 8 School of Computer Science, Duy Tan University, Da Nang, Vietnam; 9 Jadara Research Center, Jadara University, Irbid, Jordan; 10 DTU AI and Data Science Hub (DAIDASH), Duy Tan University, Da Nang, Vietnam.; Ardakan University, IRANISLAMIC REPUBLIC OF

## Abstract

Outlier detection is essential for identifying unusual patterns or observations that significantly deviate from the normal behavior of a dataset. With the rapid growth of data science, the prevalence of anomalies and outliers has increased, which can disrupt system modeling and parameter estimation, leading to inaccurate results. Recently, deep learning-based outlier detection methods have gained significant attention, but their performance is often limited by challenges in parameter selection and the nearest neighbor search. To overcome these limitations, we propose a three-stage Efficient Outlier Detection Approach (named EODA), that not only detects outliers with high accuracy but also emphasizes dataset characteristics. In the first stage, we apply a feature selection algorithm based on the Boruta method and Random Forest to reduce the data size by selecting the most relevant attributes and calculating the highest Z-score of shadow features. In the second stage, we improve the K-nearest neighbors algorithm to enhance the accuracy of nearest neighbor identification in the clustering phase. Finally, the third stage efficiently identifies the most significant outliers within clustered datasets. We evaluate the proposed EODA algorithm across eight UCI machine-learning repository datasets. The results demonstrate the effectiveness of our EODA approach, achieving a Precision of 63.07%, Recall of 82.49%, and an F1-Score of 64.53%, outperforming the existing techniques in the field.

## 1. Introduction

An outcome that significantly differs from the other datasets and seems to be unsuitable is referred to as an anomaly. Hawkins defines an outlier [1] as: “A deviation from the usual pattern that raises the possibility that an unusual technique was used to create the object is known as an outlier”. Data mining includes outlier detection as a key component and has recently gained popularity. The goal of identifying outliers is to locate data objects that stand out from the majority. This task aims to identify outliers or unusual findings [2]. Current applications of the outlier detection technique include network resilience analysis, detection of intrusions, clustering categorization, medical and health care, assessment of stock networks, and the protection of credit card scams, among others [3–4]. The last few years have seen the development of a wide range of recognition of outlier techniques, including clustering-based models, distance-based models, density-based models, probabilistic models, etc. [5]. Several deep recognitions of outlier strategies have also been presented [6–7] in addition to these shallow systems.

Outlier detection techniques are frequently used in a variety of data mining applications, including data cleaning, recognition of credit card fraud, intrusion detection for networks, web service QoS forecasting, DRAM failure estimation, and even recently trendy government legislation [8]. In recent years, the study on outlier detection has drawn the attention of researchers. Recently, several Outlier detection algorithms have been proposed based on different classifications, such as supervised, unsupervised, and semi-supervised techniques. When a training dataset already has prior knowledge about the category that every single instance falls under—normal or abnormal—supervised outlier detection is concerned. When training data are assumed to be entirely normal instances, one class support vector machines (OCSVM) or support vector data descriptions (SVDD) [9,10] carry out a hypersphere within the normal data and use the created hypersphere to identify an unidentified sample as an inlier or outlier. In numerous practical scenarios, the trained outlier detection problem presents a challenging case because obtaining label details for the whole training dataset can be costly, time-consuming, and subjective.

Unsupervised machine learning outlier detection, which does not require knowledge of the class distribution beforehand, can be broadly divided into four categories: distribution-based [11], distance-based [12], density-based [13], and clustering-based [14] methods. While anomalies are not consistent with the algorithm, the distribution-based technique believes that every data point is the result of a specific statistical model. The supervised learning-based approaches now in use have two major limitations, the first of which is the time-consuming requirement for labeled training datasets in real-time applications. Second, it fails to identify new categories of unusual events. A method based on unsupervised learning, however, does not need a labeled training dataset. It can identify the types or categories of unusual events. According to the classification, the difficulty in identifying the local density issue is a barrier to the distance-based strategy. Furthermore, it is still difficult to identify low-density patterns using density-based methods.

This paper proposes an effective method for identifying outliers that explicitly addresses the limitations of unsupervised methods. This proposed method eliminates irrelevant attributes present in the datasets by examining relevant features of the attributes and using K-nearest neighbors (KNN) search; the proposed approach uses unsupervised learning to create class-based clusters. The primary goal of the proposed study is to use data reduction techniques to remove irrelevant attributes present in the dataset and reduce the size of the dataset in its first stages. Each attribute in the dataset contributes significantly to the outlier detection process. Therefore, removing irrelevant attributes from the dataset significantly impacts how well the proposed method performs. The KNN search algorithm is used in the proposed approach to group similar class clusters based on their distance from each other. The proposed strategy also computes the degree of each point and finds outliers in the clustered dataset. The originality of this research resides in the method of training the dataset by eliminating redundant attributes, creating class-based clusters, and then identifying outliers within these clusters. The emphasis is on preparing the data by removing extraneous features, which ultimately enhances the accuracy and effectiveness of outlier recognition.

### 1.1. Our motivation

Outlier detection, particularly in large datasets, can be incredibly challenging due to the complexity of the data. As datasets grow, they often contain a mix of both useful and irrelevant features, making it difficult to accurately identify patterns that truly stand out. These irrelevant characteristics can negatively affect the precision and efficiency of outlier detection methods, leading to misleading or erroneous results. Another major challenge is accurately identifying the nearest neighbors, especially in high-dimensional data. Many existing outlier detection methods struggle with this, and if the nearest neighbors are chosen incorrectly, it can cause the entire clustering process to break down, leading to incorrect identification of outliers. Moreover, many current approaches don’t compute the Z-score and the degree of each point simultaneously, which is essential for understanding how far an observation deviates from the norm. Without this, it is harder to determine the true significance of potential outliers. Given these challenges, our work is motivated by a clear need to improve outlier detection by addressing the issue of irrelevant data, ensuring more accurate neighbor selection, and providing a more comprehensive analysis of each data point.

### 1.2. Our contributions

In this paper, we introduce EODA (Efficient Outlier Detection Approach) to tackle the mentioned challenges and provide a more reliable way of identifying outliers in complex datasets. The summary of our main contributions is as follows:

- *Proposing a three-stage outlier detection method*: One of the key innovations in this work is the integration of Boruta-Random Forest (RF) feature selection, KNN clustering, and outlier detection, allowing us to not only identify outliers but also understand the structure of the clusters they deviate from. The proposed EODA algorithm combines feature selection, improved KNN, and simultaneous calculation of both Z-scores and data point degrees. This three-stage approach enables us to detect the most significant outliers within clustered datasets, making the process more effective and efficient.
- *Reducing irrelevant data using Boruta-RF feature selection*: In the first phase of the proposed EODA algorithm, we streamline the dataset by using Boruta-RF to identify the most relevant features. By focusing only on the attributes that matter, we remove noise and redundancy, which significantly improves both the accuracy and the speed of the outlier detection process.
- *Enhancing nearest neighbor accuracy in clustering*: In the second phase, we address the problem of nearest neighbor selection. We improve the traditional KNN algorithm by using the Euclidean distance metric to find the closest neighbors more precisely. This ensures that we form accurate clusters, which is crucial for identifying outliers with confidence.
- *Proving the effectiveness across real-world datasets*: Finally, we tested our method on eight UCI datasets and compared its performance against five existing outlier detection algorithms including LOF [15], CBLOF [16], LDCOF [17], DBSCAN [18], and H-DBSCAN [19]. The results demonstrate that the proposed EODA algorithm consistently outperforms the existing methods in terms of precision, recall, and F1 score.

### 1.3. Organization

The remainder of the paper is divided into five sections: Section 2 describes the related works of the current unsupervised outlier identification system, which summarises the research on density-based techniques that have been proposed over the years. Section 3 discusses the proposed framework of outlier detection to address the issues of data reduction, searching for the nearest neighbor, and computing outlier detection along with the degree of each point. Section 4 presents the results of outlier detection over eight UCI datasets and discusses each obtained result. Section 5 focuses on the conclusion of the research paper and its future direction.

### 2. Related work

**Fig 1:**
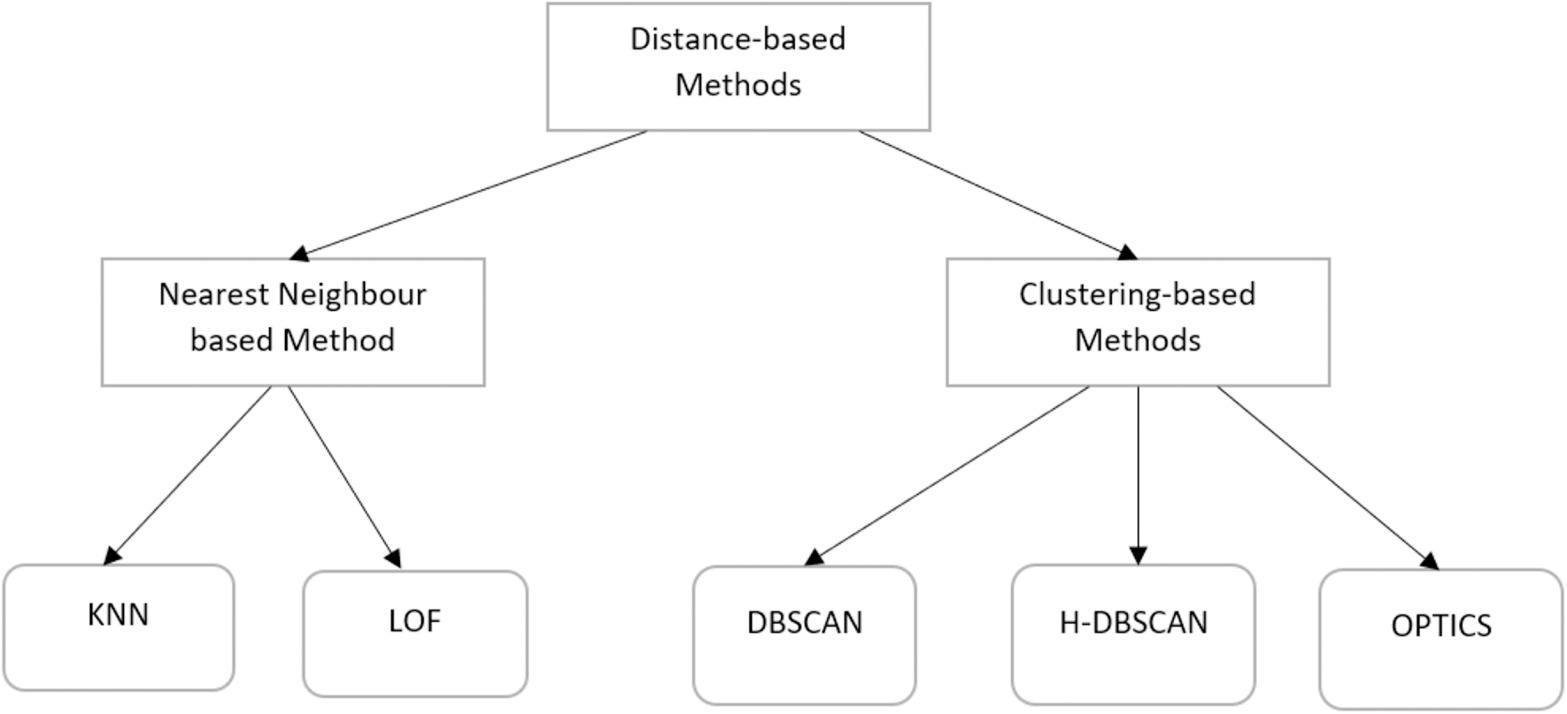
Comparative analysis of distance-based methods for outlier detection.

The recent papers on outlier detection are reviewed in this section. Several published studies on outlier detection have had an impact on our research. This section explores various topics such as the classification of outlier methods, different types of techniques used to identify outliers based on cluster-based techniques, and density-based outlier detection techniques. The efficiency of the models for identifying outliers may be impacted by the outliers. As a result, the outliers in the dataset must be eliminated to improve the performance of such models. A comparative analysis of distance-based methods for outliers’ detection is shown in Fig 1.

Existing techniques for outlier detection fall into the following categories: The following techniques are used: statistical, ensemble, distance-based, density-based, clustering-based, learning-based. Statistics-based methods are employed to draw out information from the underlying data. These methods deal with the issues presented by certain distribution points that do not fit the model. These methods, however, may not be effective when dealing with high-dimensional data adaptation caused by data points connected to several unknown distributions. An observation is considered an outlier if its immediate neighborhood has a low population density.

Distance-based outliers are identified by taking into consideration the distance to their neighbors. An adequate distance and the right number of neighbors are identified by analyzing the observation’s distance from its neighbors to predetermined threshold values. Two points are referred to as neighbors to detect outliers if their distances fall outside of a predetermined range. A parameter that is that depends on the choice of neighboring points affects the determination of an outlier. The specifications for detecting anomalies in the dataset are greatly influenced by the parameter [11]. If an object x ∈ X meets following conditions for a particular dataset X, it is referred to as an outlier:


$$\left\{\;x^{\prime }\in X|dist(x,\;x^{\prime }\;)>\;\delta |\;\geq \;\alpha n\right\}$$ (1)

where *n* represents the number of objects in the data collection, and α, δ ∈ R (1≥α≥0) stand for the threshold values.

A distance-based strategy utilizing KNN for outlier detection is developed [20] to solve the drawback of outlier ranking. By calculating the distance to each item’s kth nearest neighbor, this method determines the Z-score for each object. The proposed approach has demonstrated impressive effectiveness in outlier detection while preserving minimal computational complexity.

### 2.1. Density based outlier detection

Breunig et al. [21] developed the idea of the local outlier factor (LOF) to combine with the idea of relative density in identifying outliers. By calculating the degree of each point in relation to its neighbors, LOF evaluates KNN outlier detection and distance-based determination of outlier’s algorithms [22–29] and [30]. To assess whether a data point is abnormal, LOF considers the density of nearby points in the nearby region. Data points with higher LOF scores have a higher chance of being outliers. To find the top-n local outliers, Jin et al. [31] presented an approach since outliers frequently take a small portion of a dataset. As they are more likely to reflect the most significant outliers in the dataset, this model concentrates on choosing data points with the highest LOF scores. Additionally, several techniques [32–35] have been developed in the literature already in existence to find local outliers in static datasets. These techniques seek to detect and examine outliers in particular local dataset regions.

Tang et al. [36] proposed a method based on the connectivity-based outlier factor (COF) method, which seeks to ascertain the relative connectivity amongst data points. To determine the level of outlier-ness for each data point, this method applies the KNN methodology. The COF approach offers a trustworthy way to find outliers in a dataset by taking into consideration the closeness and connection of points. The local distance-based outlier factor (LDOF) approach has been proposed [37]. This variant calculates the local density of the data points by averaging the distances between each data point and its k-nearest neighbors. The outlier factor is then generated by estimating the relative density using the KNN method. The LDOF approach is an efficient way to find outliers in a dataset which takes advantage of both local density and relative density. A more economical variation of LOF has been published by Erich Schubert et al. [35], which uses the k-distance measure to assess the density of data points rather than the achievable distance employed in conventional LOF. However, this adjustment might provide inliers with lesser reliable outcomes. This method entails calculating the distances between data points to evaluate their density and specify the degree of irregularity for each data point. The precision of identifying inliers may be compromised by using this method, but it allows for an effective assessment of the density and irregularity of data points.

The LOF algorithm is a well-known technique for outlier detection and was first introduced by Angiulli and Pizzuti in 2002 [38]. Each data point’s neighborhood density is calculated using the LOF technique. The LOF technique provides useful insights about a data point’s local density in comparison to its surrounding data points by assessing the density of a data point concerning its neighboring points. Based on how far outliers deviate from the regional patterns of densities, this method has shown to be useful for detecting anomalies.

This work uses the LOF estimator, developed by Dragoljub Pokrajac et al. [23], to calculate the local density deviation of a given sample with nearby data points. It measures how much the density of a particular sample varies from the nearby data points, giving important information on the anomalous status of the sample. According to our method, an outlier is a sample point with a density that is noticeably lower than the sample points nearby. This description implies that the outlier is an observation that differs from the predicted density patterns displayed by its neighbors. The $LOF_{k}$ measure is expressed as per the following:


$$LOF_{k}\left(x_{i}\right)=\;\frac{1}{k}\;\left(\sum {\mathrm{\backslash nolimits}}_{x_{l\;}\in \;N_{k}\left(x_{i}\right)}\sum k-dist\left(x_{l}\right)\right)\;\left(\sum {\mathrm{\backslash nolimits}}_{x_{j\;}\in \;N_{k}\left(x_{i}\right)}frac1\sum_{x_{m\;}\in \;N_{k}\left(x_{j}\right)}k-dist\left(x_{m}\right)\right)$$ (2)

where the measurement of the distance between a data point’s k nearest neighbors, or the set $N_{k}\left(x_{i}\right)$, and that point’s $x_{i}$ can be generally referred to as the phrase $k-dist\left(x_{i}\right)$. It estimates the separation between $x_{i}$ and the edge of the data point’s immediate neighborhood and provides important details about the local density features of the data point.

The COF algorithm, which was developed by Tang et al. [39], aims to identify underlying data patterns. To determine how many neighbors are closest, the COF algorithm uses the set-based nearest (SBN) method. An in-depth analysis of the local density features in the dataset becomes feasible using the SBN route within COF to find and calculate its nearest neighbors for each data point. Additionally, the average chain distance of the experiment site is taken into consideration when using the SBN technique to compute the relative density. By comparing the test point’s density to those of its surrounding data points, this calculation aids in determining the test point’s local density characteristics. The relative density distributions are examined in the LOF and COF techniques to identify outliers. The LOF algorithm has been significantly enhanced by Jin et al. [40], who have also unveiled INFLO, a brand-new density-based outlier detection technique. The core concepts of LOF are expanded upon in INFLO, which also incorporates enhancements to increase the precision and efficacy of outlier detection using density analysis. The influenced space, which is created by merging an object’s neighboring data points and reverse neighboring data points, is used in the INFLO method to estimate relative density. This extensive method considers the influence of both the object’s neighbors and its reverse neighbors, allowing for a more accurate calculation of the relative density. Each data point’s connectivity pattern with its neighbors is the focus of the COF algorithm. The COF algorithm offers useful insights into the connections and interactions among data points by focusing on the connectivity element, allowing for a more in-depth examination of outlier detection based on density characteristics. This connection pattern is measured using a score represented by the following ratio:


$$COF_{k}\left(p\right)=\;\frac{\left|N_{k}\left(p\right)\right|ac-dist_{N_{k}\left(p\right)}\;\left(p\right)}{\sum_{o\;\in \;N_{k}\left(p\right)}ac-dist_{N_{k}\left(o\right)}\;\left(o\right)}$$ (3)

where $ac-dist_{N_{k}\left(p\right)}$ is the average chaining distance between point p and its k-1 nearest neighbors in the set $N_{k}\left(p\right)$, which is defined as $ac-dist_{N_{k}\left(p\right)}\;\;\;\;\;\;\;\left(\text{p}\right)=\;\;\;\;\;\;\;\sum {\mathrm{\backslash nolimits}}_{i=1}^{k-1}\frac{2\;\;\;\;\;\;\;\left(k-i\right)}{k\;\;\;\;\;\;\;\left(k-1\right)}dist\left(e_{i}\right)$, where $e_{i}$ stands for the edges used in the calculation. The average distance between point *p* and its *k*-1 nearest neighbors is measured by the $ac-dist_{N_{k}\left(p\right)}\;\;\;\;\;\;\;\left(\text{p}\right)$, which is a key parameter in some outlier detection algorithms as well as analysis.

### 2.2. Clustering-based outlier detection

The primary objective of clustering algorithms, on the other hand, is to uncover patterns that classify the fundamental information and group it into discrete clusters, distinguishing between normal clusters and outliers that vary from typical clusters. Clustering-based unsupervised outlier detection techniques have drawn a lot of interest and have grown in popularity [41]. These methods are beneficial as they create detection models without the need for labelled training data, enabling them to be particularly helpful in circumstances when labeled data is scarce or unavailable. The benefit of clustering-based techniques in the outlier detection problem is their applicability in situations where acquiring labeled data or perfectly regular data is difficult.

#### 2.2.1. K-means clustering method.

Partitional and hierarchical methods can be generally used to categorize clustering methods. Partitioning techniques, like distance-based methods, use distance measures (such as Euclidean or city block distances) to compare the similarity of data points and group them into clusters according to predetermined standards. While identifying clusters, density-based algorithms evaluate the density of data regions. By repeatedly integrating or dividing clusters, hierarchical clustering creates a hierarchy of clusters. Depending on the data properties and desired level of control during the clustering process, one may choose between partitional and hierarchical clustering [24,25]. The Euclidean distance, which is used in Eq (6), is a popular option for methods of clustering because it accurately expresses geographic relationships between data points.


$$X=\left(x_{1},\;x_{2},x_{3},\ldots \ldots ..,x_{n}\right)$$ (4)

$$M^{r}=\left(m_{1}^{r},\;m_{2}^{r},\;m_{3}^{r},\;\ldots \ldots .,m_{n}^{r}\;\right)$$ (5)

$$x_{l}\;\in \;C_{i}^{r}:dist\left(x_{l},m_{i}^{r}\right)\;\leq {\{dist\left(x_{l},m_{j}^{r}\right\}}^{k},\text{j}=1,\text{ j}\neq i\;,\;1\leq i\;\leq k\;,\;x_{l}\in X$$ (6)

The 2-dimensional samples can be used to express by Equation (7) using the Euclidean distance: $x_{i}=\left(f_{1}^{i},\;f_{2}^{i},\ldots ..,\;f_{d}^{i}\right)$ and $\left(f_{1}^{j},\;f_{2}^{j},\ldots ..,\;f_{d}^{j}\right)$ as


$$ED={\left(\sum {\mathrm{\backslash nolimits}}_{l=1}^{d}{\left(f_{l}^{i}-f_{l}^{j}\right)}^{2}\right)}^{1/2}$$ (7)

Additionally, as shown in Equation (8), new centroids are identified by modifying $M^{r}$ at every repetition. The K-means technique can be terminated if the constituents of $C_{i}^{r}$and $C_{i}^{r+1}$ remain unchanged during subsequent iterations.


$$m_{i}^{r+1}=\;\frac{1}{\left|C_{i}^{r}\right|}\sum {\mathrm{\backslash nolimits}}_{x_{j\in C_{i}^{r}}}x_{j}\;1\leq i\;\leq k$$ (8)

#### 2.2.2. K-Nearest neighbor method.

The KNN algorithm, a popular regression and classification technique, is employed to identify the closest neighbors for a given sample, considering relevant sample measurements. For a given dataset D consisting of k clusters with similar member labels (*p*), the KNN algorithm can be utilized to determine the nearest neighbors for a test sample $TS_{h}$ from a test set (TS). To achieve this, the distance between the test sample $TS_{h}$ and the cluster members $x_{ji}$ is calculated using Equation (9), and it is denoted as d_ji. Next, the values of $d_{ji}$ are sorted in ascending order to create $ds_{ji}$ as shown in Equation (10). The average of the K members from $ds_{ji}$ is then calculated as 𝑎𝑣𝑒_𝑖_, which represents the best value of K according to the model described in Equation (11). The outlier detection within $TS_{h}$ is based on the cluster with the least 𝑎𝑣𝑒 value, as determined by Equation (12).


$$d_{ji}=dist\;\left(TS_{h},\;x_{ji}\;\right)\;h=1,\;2,\;3,\;...,\left|TS\right|,\;j=1,\;2,\;\ldots ,\;\left|\;C_{i}\right|\;i=1,\;2,\;\ldots ,\;p$$ (9)

$$ds_{ji}=sort\left(d_{ji}\right)$$ (10)

$$ave_{i}=\;\frac{1}{K}\sum {\mathrm{\backslash nolimits}}_{j=1}^{K}ds_{ji}$$ (11)

p=min(ave)  (12)

Although KNN is frequently used as a classification approach, in this case, it has been employed to determine distance. Support vector machine (SVM) and RF have been applied on two-labeled and multi-labeled datasets, respectively, to assess the efficacy of the methods. A linear kernel function with a 2-box restriction was used by SVM in this work. Instead of using just one decision tree, the RF approach used many single decision trees, which resulted in more accurate results.

An extensively researched technique for identifying outliers is the CBLOF (clustering-based LOF) algorithm [42]. It has been introduced by He et al. in 2003 [42]. By taking into consideration the properties of data clusters, the algorithm concentrates on locating local outliers. Using a clustering method like k-means or DBSCAN, the CBLOF algorithm divides the data into clusters. Then, a score is given to each cluster depending on the size and average distance between the data points within the cluster. Each data point’s LOF, which reflects how out of the ordinary it is compared to its nearby cluster, is determined. The LOF is determined by comparing the density of the data point to the densities of its neighbors. The CBLOF algorithm offers a computationally efficient approach for outlier detection, as it only considers the size of clusters and does not require calculating the exact density of each cluster. It has been applied in various domains, such as fraud detection, intrusion detection, and network traffic analysis, where identifying local anomalies within clusters is crucial. Further research and literature reviews on the CBLOF algorithm can provide more in-depth insights into its theoretical foundations, variations, and experimental evaluations.

In the LDCOF (local density cluster-based outlier factor) algorithm, the data points are grouped into clusters based on their similarities using clustering techniques such as k-means, DBSCAN, or hierarchical clustering. Each cluster’s local density is calculated by considering the distances between the data points within the cluster. To determine the outlier factor for each data point, the LDCOF algorithm assesses the density of the data point concerning its neighboring points and the density of its corresponding cluster [43–47]. A higher outlier factor indicates that a data point has a lower density compared to its neighbor’s and the density of its cluster, suggesting its potential outlier status. By combining local density and clustering information, the LDCOF algorithm aims to provide a more comprehensive and accurate outlier detection approach. It can be applied to various domains and datasets where understanding local density patterns and cluster structures is crucial for identifying outliers. However, further literature or references regarding LDCOF are needed for a more H-DBSCAN (hierarchical density-based spatial clustering of applications with noise) is an extension of the DBSCAN algorithm that incorporates a hierarchical approach to clustering. While there may not be a specific algorithm known as “H-DBSCAN” in the literature, there are variations and related works that incorporate hierarchical clustering with density-based techniques. The density-based clustering and hierarchical approaches hybrid HDBSCAN algorithm were introduced in [48–51]. It is possible to find clusters of various densities and sizes using HDBSCAN, which builds a hierarchical cluster tree. It deals with the shortcomings of the conventional DBSCAN method.

Several well-known outlier detection methods have been assessed in this section, and it has been noticed that many of them rely on the same model parameter, k (neighborhood size). Determining whether k is used simultaneously with the distance-based or density-based strategy is essential since it has a significant impact on the efficiency of outlier detection. We describe a unique outlier detection technique that makes use of the idea of natural neighbors to get around these restrictions. A summary of comparison analysis of the existing research for outliers’ detection methods is discussed in Table 1.

**Table 1:** Summary of comparison analysis of the existing studies for outlier detection.

Authors	Year	Technology Used	Description	Advantages	Limitations
Smith et al. [49]	2014	Dimensionality reduction and kernel density	Two-stage online anomaly detection algorithm using dimensionality reduction and kernel density estimation as non-conformity measures	- Captures essential features of the data	- Limited to time series data
Schubert et al. [35]	2014	Angle-based outlier detection	Outlier detection based on angle-based outlier detection	- Robust to noise and able to handle high-dimensional data	- Requires setting a suitable threshold
LV et al. [45]	2016	Robust PCA	Outlier detection using robust principal component analysis	- Robust to outliers and resistant to noise	- Limited to linear relationships
Tang et al. [28]	2017	Feature Selection and Clustering	Outlier detection using feature selection and clustering techniques	- Identifies meaningful data relationships - Enhances detection accuracy	- Requires determination of appropriate
Gyoung et al. [25]	2018	Feature Selection and Clustering	Outlier detection using feature selection and clustering techniques	- Captures essential information - Improves anomaly detection performance	- Requires determination of appropriate
Zhang et al. [41]	2018	Feature Selection Clustering	Outlier detection through feature selection and clustering techniques	- Reduces dimensionality and focuses on relevant features	- May lose important information in feature reduction
Li et al. [50]	2018	Feature Selection and Clustering	Outlier detection using feature selection and clustering techniques	- Focuses on relevant features	- Requires prior knowledge or assumptions
Wang et al. [34]	2021	Robust feature matching	Matching of robust features using LOF	- User defined threshold used to classify the mismatches	- mismatches with the error-prone local feature detectors
López-Oriona et al. [37]	2021	A functional data approach	Outlier detection for multivariate time series	-Robust to outliers and resistant to noise	- Increased computational complexity
Huang et al. [51]	2021	Outlier Ensembles	Outlier detection using ensembles of clustering algorithms	- Combines results from multiple clustering approaches	- Increased computational complexity
Hilal et al. [27]	2022	Supervised and unsupervised	Outlier detection using semi supervised and unsupervised methods	- Employs statistical, artificial intelligence and machine learning models for more accurate outlier detection	- Sensitive to selection hyperparameters
Proposed Work	2024	Data reduction using Boruta algorithm & clustering of datasets using KNN	An effective outlier detection for data reduction through feature selection and clustering techniques	- Obtain the reduced datasets - Detection of outliers based on reduced datasets	- Noise in datasets may affect the accuracy

### 2.3. Problem definition

The purpose of this research is to create an efficient feature selection and clustering approach for outlier detection. Outliers, which dramatically differ from the rest of the data, might offer insightful information or hint at problems with the quality of the data. However, because the data is so complicated and has a high degree of dimensionality, finding outliers in large datasets can be difficult. The primary issue to deal with is feature selection: Find the most relevant characteristics for outlier detection by developing a feature selection strategy [52–55]. The subset of attributes that can most effectively distinguish between typical data and outliers should be automatically identified by the system.

- *Clustering Techniques*: The work requires exploring and using clustering methods to group comparable data points together. These algorithms examine the underlying structures and patterns in the data to find clusters and separate outliers from typical data points.
- *Outlier Detection*: Create an outlier detection system that makes use of the selected attributes and clustering outcomes to locate and highlight probable outliers. For each data point, the approach should assess the degree of abnormality by considering both the feature values and the distance from cluster borders.

This paper proposes a stable and effective strategy for outlier detection in big and complicated datasets by combining feature selection and clustering. The proposed EODA approach has the potential to be used in a variety of fields such as banking, healthcare, fraud detection, and detection of anomalies in industrial systems. In Section 3, the proposed EODA model is introduced to accomplish the research paper’s objectives. Using feature selection and clustering approaches, the EODA model was created to overcome the difficulties associated with the existing outlier detection techniques.

## 3. Proposed EODA outlier detection algorithm

**Fig 2:**
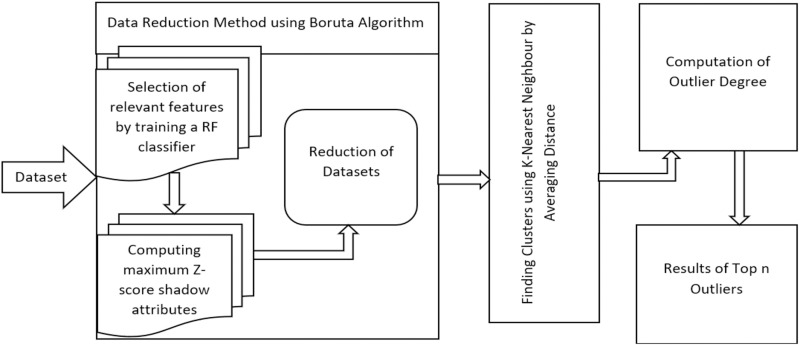
The proposed algorithm of the outlier detection method.

To achieve high accuracy and efficiency when mining outliers in high-dimensional datasets, there are multiple challenges to overcome. When used to mine for outliers in these datasets, existing techniques frequently have issues with the effectiveness of the outlier method. To address this problem, we provide a data reduction strategy that concentrates on reducing the dimensions and data points before executing outlier identification methods. In this section, the process of eliminating irrelevant attributes from the dataset is outlined in Section 3.1. The technique explores cluster-exhibiting dimensions in the dataset, enabling us to locate dense clusters and their positions in each dimension. The objective is to distinguish between dimensions that matter and those that don’t for outliers. Performing this, we can locate a subspace with dense points in irrelevant dimensions. By reducing the number of sparse regions, the efficiency of data clustering has also been improved using this attribute relevance analysis idea. In the methodology section, Fig 2 provides a description of the proposed scheme architecture for finding outliers in huge datasets. This scheme works in three stages as follows:

- **Stage 1: Feature Selection**: The objective of this stage is to select the high-dimensional dataset’s most significant and instructive features. The significance of each feature can be assessed using feature selection approaches like mutual information, correlation analysis, or recursive feature reduction. We can decrease dimensionality to raise the precision of outlier detection by choosing effective features that accurately depict the fundamental trends in the data.
- **Stage 2: Clustering:** Clustering methods are used to combine data points that are associated after the relevant attributes have been recognized. You can use a variety of clustering methods, including k-means, hierarchical clustering, and density-based clustering. The idea is to divide the dataset into discrete clusters, where each cluster stands for a collection of data points with a common set of attributes. Clustering makes it easy to spot outliers that do not fit into any cluster by identifying the regular behaviors that occur within the data.
- **Stage 3: Outlier Detection**: The next phase is to find outliers within the dataset after collecting the clusters. Considering the difference or differing characteristics between each data point and its associated cluster will help with this. Potential outliers can be data points with a large divergence from the specified cluster centroid, a large distance from it, or low density in the grouping region. To further detect outliers based on how they differ from the data’s usual distribution, statistical techniques like the Z-score or Mahalanobis distance can be computed.

The proposed approach follows a structured three-stage process to enhance outlier detection. In Stage 1: Feature Selection, the method identifies the most significant and informative features from high-dimensional datasets. Techniques such as mutual information, correlation analysis, or recursive feature reduction are utilized to retain features that capture key trends and variations. This step reduces dimensionality, improves computational efficiency, and enhances the precision of outlier detection. In Stage 2: Clustering, the selected attributes are used to group data points into clusters using k-means, hierarchical clustering, or density-based clustering. These clusters represent groups of data points with similar characteristics, allowing for identifying regular patterns within the data. In Stage 3: Outlier Detection, the deviations of individual data points from their respective clusters are analyzed. Outliers are identified based on significant divergence from cluster centroids, large distances from cluster boundaries, or low-density regions. Statistical techniques such as the z-score or Mahalanobis distance further refine this process, enabling precise and reliable detection of anomalies within the dataset.

### 3.1. Elimination of irrelevant attributes

The process of developing predictive models needs to incorporate the feature selection process since it aids in determining which features are most useful for forecasting the target variable. Outliers in the dataset, however, may affect the importance ratings of the features, which may then impact the selection procedure. The Boruta algorithm is a feature selection approach created to solve the drawbacks of conventional techniques that depend upon statistical hypotheses that might not hold in datasets from the real world.

The first phase of the Boruta method generates “shadow” properties for every variable. The original features are permuted arbitrarily to produce these shadow qualities, which eliminates biases and correlations between them. To train an RF classifier, which determines the Z-scores indicating the significance of each feature, this expanded information system is subsequently utilized. The technique is iteratively performed to produce statistically significant outcomes, and the maximum Z-score amongst the shadow characteristics (abbreviated as MZSA) is established for each iteration.

The Z-scores for each of the actual attributes are analyzed and compared to the MZSA. Any attribute that has a Z-score below the MZSA is labeled “Unimportant” and is then deleted from the database. On the other hand, each attribute that has a Z-score greater than the MZSA is labeled as “Important.” This procedure is repeated until all attributes are classified as “Important” or “Unimportant,” or until a predetermined number of RF iterations have been completed. The final result of the Boruta method then returns the features that have been selected after the shadow characteristics have been eliminated.

**Algorithm 1. Feature Selection Method for Removing Irrelevant Attributes**

**Inputs:** Feature Matrix and Target Matrix of Original Data, maximum number of iterations

**Output:** Removal of the irrelevant features and selecting the relevant features from datasets

**1. INITIALIZATION:**

2. x⇓Original Features

3. y⇓Shadow Features

4. R⇓Origin

5. M⇓ Feature Matrix of Original Data

6. N⇓ Target Matrix of Original Data

7. n⇓maximum number of iterations

8. S⇓New Matrix

9. D⇓Dataset

10. $Z_{Orinal\_score}$⇓Z_Score of Original Features

11. $Z_{Max\_score}$⇓Maximum Z_Score of Shadow Features

12. **BEGIN:**

13. for (i = 0; i < n; i++)

14. P⇓Generate(Matrix of Shadow Features)

15. S⇓Fit_Model(P,R)

16. if ($Z_{Orinal\_score}>\;Z_{Max\_score}\;$)

17. D⇓ Assign (x)

18. else

19. Removal of the original features from the dataset

20. end if

21. End for

22. Compute average gain of the selected original features.

23. **END**

The proposed method assigns each attribute a relevance score depending on how closely it relates to the target variable. Then, it resolves redundancy across each of the selected features and identifies the relevant attributes above a predetermined level. The result is a dataset that has been condensed to just include the crucial elements and is prepared for additional simulation or assessment.

The novelty of this algorithm lies in its integration of advanced feature selection techniques, such as mutual information or recursive feature elimination, with the specific aim of optimizing outlier detection. By focusing on identifying and removing irrelevant attributes, the algorithm significantly reduces the dimensionality of the dataset while preserving the core information required for accurate analysis. This step is particularly innovative in the context of outlier detection, as it ensures that the detection process is both computationally efficient and precise.

### 3.2. Searching the nearest neighbors

The KNN algorithm is a supervised learning algorithm commonly used for classification and regression tasks. It operates by identifying the k nearest neighbors to a given data point in the feature space and utilizing their labels or values to make predictions. While the KNN algorithm itself does not explicitly handle outliers, it can be employed as a tool for outlier identification and handling within a dataset. This can be achieved by using the KNN algorithm to calculate the distances between each data point and its k nearest neighbors. If a data point is significantly farther away from its k nearest neighbors compared to the rest of the data points, it can be classified as an outlier. These outliers can then be managed by either removing them from the dataset or treating them differently, depending on the specific analysis requirements. Additionally, the KNN algorithm can be utilized for imputing missing values in a dataset. This involves using the KNN algorithm to identify the k nearest neighbors for each data point with missing values and then using the average (for numerical data) or mode (for categorical data) of the corresponding values from the nearest neighbors to impute the missing values.

The algorithm improves upon the traditional KNN search by incorporating enhancements tailored for clustered datasets. Its novelty lies in dynamically adjusting the neighborhood search based on the clustering structure, which mitigates the impact of high-dimensional noise and improves the identification of nearest neighbors. This approach allows for more accurate clustering and subsequently enhances the precision of outlier identification in the next stage.

**Algorithm 2. KNN Search Algorithm for Cluster Dataset**

**Inputs:** Dataset as X = {$x_{1}$, $x_{2}$, $x_{3}$, ………, $x_{i}$} where $x_{1}$, $x_{2}$, $x_{3}$, ………, $x_{i}$ are the data points of clusters to identify the class, Threshold Value as Thresh_k, Nearest Neighbors C: {$c_{1}$, $c_{2}$, $c_{3}$, ………, $c_{i}$}

**Output:** Class of Clusters as $c_{x}$ where $c_{x}\in C$, x = 1,2,3,……

**1. INITIALIZATION:**

**2.** K⇓3

**3.** m⇓len (C)

**4. BEGIN:**

**5.** for (j = 1; j<=m; j++)

**6.** Compute Euclidian_Distance (j, $x_{i}$})

**7.** End for

**8.** for (K = 3; K<=Thresh_k; K++)

**9.** for ($c_{x}$= 1; $c_{x}$<=m; $c_{x}$++)

**10.** if (K_nearest < k && Euclidian_Dist  >= Euclidian_Dist_Ordered)

**11.** Take the data points from x

**12.** End if

**13.** End for

**14.** End for

15. Compute the class of the x:$c_{x}=argmax\sum {\mathrm{\backslash nolimits}}_{y\in N}I\;\left(c=class\;\left(y\right)\right)$

16. **END**

### 3.3. Detection of outlier instances

Data points that greatly depart from the rest of the data and display anomalous behavior are those that need to be identified to be classified as outlier cases. Detecting abnormalities or pointing out problems with data quality, outliers offer insightful information. The LOF and KNN algorithms, which evaluate distance or density between data points, are examples of distance-based methods. The term “outlier” refers to a point with exceptionally high or low density.

The proposed algorithm combines feature selection, clustering, and outlier detection techniques to identify and classify outlier instances in the dataset. This code represents an outlier detection algorithm. It takes as input a set of clusters C obtained by applying another algorithm (Algorithm 2). For each cluster i in C, it calculates the density of instances within that cluster by summing the distances of each instance to all other instances in the cluster. Then it calculates an outlier score for each instance in the cluster by multiplying its density by its distance to the nearest higher density instance. Alternatively, it can use the inverse of distance instead of distance in the outlier score calculation. It then merges each cluster of probable outliers and computes the set of non-outlier instances N_pts by subtracting the probable outlier instances from the original data set D. Finally, it sorts the outlier scores in descending order and generates the top-N outliers.

The proposed outlier detection algorithm exhibits novelty in its multi-faceted approach to isolating outliers. By combining statistical methods, such as z-scores or Mahalanobis distance, with cluster-based anomaly analysis, the algorithm addresses both local and global variations in the dataset. Its ability to analyze deviations within clusters while simultaneously considering inter-cluster relationships provides a comprehensive framework for outlier detection. This integration of techniques distinguishes it from existing methods and underscores its innovative contribution to the field. The combination of the Boruta-RF feature selection method, enhanced KNN clustering, and the proposed outlier detection approach, is rooted in the complementary strengths of each component. Boruta-RF effectively identifies the most relevant features, improving the accuracy of the subsequent clustering process by reducing dimensionality and eliminating irrelevant noise. Clustering then groups data points with similar characteristics, which facilitates the detection of outliers based on their deviation from the typical patterns. This unified framework highlights how each stage contributes to the overall performance, demonstrating that the combination of these techniques leads to more accurate and robust outlier detection.

**Algorithm 3. Proposed EODA Algorithm for Detection of Outlier Instances**

**Inputs:** Dataset as D, Set of Clusters as C

**Output:** Instances of Outliers, Set of Outlier Clusters

**1. INITIALIZATION:**

2. C⇓{$c_{1}$, $c_{2}$, $c_{3}$, ………, $c_{i}$}// Set of Clusters after applying algo. 2

**3. BEGIN:**

4. for (i=1; i<=$c_{i}$; i++)

5. $density\left(i\right)=\;\sum {\mathrm{\backslash nolimits}}_{k\neq i,\;k\in c_{i}}X\;\left(d_{ik}-d_{c_{i}}\right)$// $d_{ik}$ is the instance distance to other instance distance present within clusters$c_{i}$

6. Compute the nearest distance from the higher-density instances

7. Calculate outliers’ instances as:

$Probable_{Outliers\_Score}\left(i\right)$ ⇓ density(i)*distance (i) or$density\left(i\right)*\;\frac{1}{distance\;\left(i\right)}$

8. End for

9. Merging each cluster of probable outliers.

10. Compute $N_{pts}$ ⇓ D - $Probable_{Outliers\_Pts}$.

11. Compute $OD_{list}$ ⇓ Sort ($OD^{\prime }-descending^{\prime }$).

12. Generate Top-N outliers.

13. **END**

### 3.4. Computational complexity analysis

The computational complexity of the proposed EODA is derived by analysing its three main algorithms: feature selection, KNN-based clustering, and outlier detection. Algorithm 1, the feature selection method, processes the dataset by iterating up to a predefined maximum number of iterations n. During each iteration, shadow features are generated, Z−scores are calculated, and features are evaluated for relevance. These operations yield a complexity of O(n×f), where f represents the number of features. This step is efficient for reducing dimensionality while retaining essential attributes.

In Algorithm 2, the KNN-based search algorithm computes distances between data points within clusters and identifies the nearest neighbours. The Euclidean distance computation for m clusters and i instances dominate the process, with a complexity of O(m×i×logi), including sorting operations for nearest neighbour identification. This complexity reflects the effort to balance accuracy and scalability for clustering.

Finally, Algorithm 3 calculates density values for clustered data points and identifies outliers based on their scores. For each cluster, density and outlier scores are computed with a complexity of O(i × k), where k is the average number of points per cluster. Sorting outlier scores to identify the top instances introduces an additional complexity of O(i×logi). Thus, the overall complexity of this stage is O(i×(k+ logi)).

Combining the complexities of all three stages, the overall computational cost of EODA is approximately O (n×f+m×i×logi +i×(k+ logi)). This complexity indicates that EODA is efficient for datasets of moderate size but may face scalability challenges with extremely large datasets due to the dependence on the number of instances and clusters.

## 4. Results and discussion

The purpose of this experimental research work is to assess how well the proposed EODA algorithm performs. To demonstrate the enhanced performance of our machine learning approach in outlier detection, we performed a comparison study employing eight selected datasets from the UCI Machine Learning Repository, openly available in [56] (website: http://archive.ics.uci.edu/ml/datasets). In the following, the effectiveness of our work is assessed using these real-world datasets as useful benchmarks. In our studies, we examined the results that were obtained from our proposed method with those five well-known unsupervised outlier detection methods: LOF [15], CBLOF [16], LDCOF [17], DBSCAN [18], and H-DBSCAN [19]. Based on their applicability to unsupervised learning techniques for outlier detection and their shared parameter, k, these algorithms were chosen.

The Python programming language was used to implement the EODA algorithm. The tests were carried out on a computer with a 3.40 GHz Intel(R) Core (TM) i7-4770 CPU, 6 GB of RAM, and a RAM frequency of 799.0 MHz. Table 2 displays the performance measurements that were taken into consideration and offers perspectives on multiple dimensions of the outlier detection method’s performance, including its capacity to accurately detect outliers, manage false positives, and strike a balance between precision and recall. Depending on the specific objectives and specifications of the identifying outlier’s task, the best metric needs to be chosen. Other often-used metrics include precision, recall, and F1-Score. Precision is defined as the percentage of outliers among all samples that were accurately recognized to be outliers. The fraction of accurately detected outliers among all real outliers is known as recall, also known as the true positive rate or sensitivity. Table 2 provides the performance metrics used for the evaluation of the performance of the proposed method.

**Table 2:** Metrics of performance evaluation parameters.

Performance metric	Formulation
Detection rate	$DR=\frac{TP}{\left(TP+FP\right)}$
False acceptance rate	$FAR=\frac{FP}{FP+TN}$
Accuracy	$ACC=\frac{\left(TP+TN\right)}{\left(TP+\;TN+FP+FN\right)}$
Area under curve	AUC=(1+TPR−FPR)
True positive rate	$TPR=\frac{TP}{\left(TP+FN\right)}$
F1-score	$F1-score=\frac{TP}{\left[TP+\frac{1}{2}*\left(FP+FN\right)\right]}$

The efficiency of the proposed outlier detection method has been assessed in this section using various parameters. First, the data reduction is required to be performed on the given dataset and then classified into clusters by employing the KNN method. By setting the different parameter values for the proposed method to compute the degree of each data point for identifying the outliers, precision, recall, and F1-score are secondarily calculated for analyzing the performance on 8 randomly chosen datasets which include Diab, Audiology, Hepatitis, Cred, Heart, Lymp, Grem, and Monks. A thorough case study is also used to demonstrate the logic of the proposed technique. Additionally, we have compared our EODA method with the other existing state-of-the-art techniques to demonstrate the effectiveness of the proposed method in terms of outlier identification. In this study, precision (P), recall (R), and F1-Score are used as assessment measures to evaluate the performance of the EODA method. These measures enable us to evaluate the effectiveness and performance of the EODA algorithm in outlier detection objectively. Since most outlier detection techniques produce an outlier factor value for each sample, these values can be ordered downward and given sequence numbers beginning at 1. The initial position can be indicated by setting the currently selected sequence ID as t = 1. By considering the value of the outlier factor with t, we can demonstrate if a sample qualifies as an outlier. It is important to keep in consideration that selecting t too small or too large could lead to the misclassification of normal points as outliers or missing the significance of true outliers. We use the parameters P(t) and R(t) to determine the ideal value for t to solve this problem. We can determine the relevant precision (P) and recall (R) values by adjusting t. For a given t, the precision (P(t)) is the proportion of correctly detected outliers to all samples that have been categorized as outliers. The recall (R(t)) shows the percentage of all the true outliers that have been correctly recognized as outliers.


$$\begin{array}{c} P\left(t\right)=\;\frac{\left|OS\left(t\right)\cap OS^{\circ }\right|}{\left|OS\left(t\right)\right|}\times 100\% \end{array}$$ (13)

$$\begin{array}{c} R\left(t\right)=\;\frac{\left|OS\left(t\right)\cap OS^{\circ }\right|}{\left|OS^{\circ }\right|}\times 100\% \end{array}$$ (14)

Let OS(t) be the collection of outliers identified by a certain threshold t, which may be considered as a function of t. The real set of outliers is referred to as OS. Additionally, P(t) and R(t) stand for the ratios of outliers that were successfully identified at the specified threshold t to the total number of true outliers (P(t)) and all true outliers (R(t)), respectively. The EODA technique removes the dense regions of each dataset using a data reduction approach. This approach becomes particularly significant when dealing with datasets that have many attributes, as it results in a higher number of attribute reductions. In comparison, for the Grem and Audiology datasets, the EODA approach demonstrates competitive performance slightly superior to that of CBLOF and LDCOF.

When evaluating outlier detection techniques using precision, the number of true outliers among the top-n ranked instances is considered. Consequently, the precision value can be low for datasets with a small proportion of outliers, such as Grem and Audiology. Conversely, the precision value might be high if the proportion of inliers is relatively low. To provide a comprehensive assessment of the overall performance of outlier detection techniques, a new approach called average precision (AP) has been introduced. Average precision takes into account the performance across a wide range of possible choices for n. Instead of evaluating at a single value of n, the value of precision is averaged over the ranks of all outlier points. This enables a more comprehensive evaluation of the effectiveness of outlier detection techniques. Experimental results of the proposed EODA method against existing outlier detection techniques on eight datasets are shown in Tables 3–10. The obtained results demonstrate the effectiveness of the proposed EODA algorithm compared to other existing methods across various datasets.

**Table 3:** Results of the proposed method against existing techniques on Diab dataset.

t	LOF			DBSCAN			H-DBSCAN			CBLOF			LDCOF			Proposed EODA		
**P(t)**	**R(t)**	**F1(t)**	**P(t)**	**R(t)**	**F1(t)**	**P(t)**	**R(t)**	**F1(t)**	**P(t)**	**R(t)**	**F1(t)**	**P(t)**	**R(t)**	**F1(t)**	**P(t)**	**R(t)**	**F1(t)**
15	55.42	29.84	38.8	55.37	36.68	44.13	78.43	47.92	59.5	68.78	45.54	54.8	78.27	54	63.91	78.43	47.92	59.5
24	36.64	29.84	32.9	47.28	45.54	46.4	73.28	71.34	72.3	73.28	71.34	72.3	75.84	71.34	73.53	73.28	71.34	72.3
37	45.67	63.42	53.11	42.85	54	47.79	54.62	78.42	64.4	59.54	82.36	69.12	64.37	87.45	74.16	62.67	95.54	75.7
45	39.78	78.42	52.79	34.71	63.42	44.87	45.83	82.36	58.9	53.76	93.17	68.18	50.34	95.54	65.94	57.65	100	73.14
53	47.11	79.44	59.15	38.51	71.34	50.02	47.28	82.36	60.08	48.85	93.17	64.1	54.83	100	70.83	54.83	100	70.83
56	41.25	79.44	54.31	41.29	78.42	54.1	39.55	82.36	53.44	47.18	100	64.12	47.18	100	64.12	47.18	100	64.12
89	33.44	93.17	49.22	32.56	100	49.13	33.44	93.17	49.22	32.56	100	49.13	32.56	100	49.13	32.56	96	48.63
105	40.72	100	57.88	40.72	95	57.01	27.94	93.17	42.99	40.72	97	57.37	40.72	96	57.19	40.72	100	57.88
163	23.54	100	38.11	23.54	100	38.11	23.54	100	38.11	23.54	100	38.11	23.54	100	38.11	23.54	100	38.11
Avg	40.4	72.62	48.48	39.65	71.6	47.96	47.11	81.24	55.44	49.81	86.96	59.7	51.97	89.37	61.88	52.32	90.09	62.25

**Table 4:** Results of the proposed method against existing techniques on Audiology dataset.

t	LOF			DBSCAN			H-DBSCAN			CBLOF			LDCOF			Proposed EODA		
**P(t)**	**R(t)**	**F1(t)**	**P(t)**	**R(t)**	**F1(t)**	**P(t)**	**R(t)**	**F1(t)**	**P(t)**	**R(t)**	**F1(t)**	**P(t)**	**R(t)**	**F1(t)**	**P(t)**	**R(t)**	**F1(t)**
48	57.33	47.37	51.88	54.83	43.86	48.74	67.34	54.39	60.18	63.68	56.14	59.68	77.78	66.67	71.8	75.45	64.78	69.71
115	39.44	78.95	52.61	32.97	64.78	43.7	44.56	84.67	58.4	44.56	84.67	58.4	47.17	94.77	62.99	47.17	94.77	62.99
123	34.54	82.7	48.73	30.34	71.56	42.62	36.75	84.67	51.26	36.75	84.67	51.26	45.56	96.49	61.9	45.56	100	62.6
147	37.84	87.34	52.81	32.47	87.47	47.36	32.54	85.32	47.12	32.54	85.32	47.12	37.78	100	54.85	37.78	100	54.85
185	36.64	96.68	53.15	32.67	87.34	47.56	36.64	96.68	53.15	32.56	91.23	48	35.56	100	52.47	35.56	100	52.47
191	32.97	96.68	49.18	32.69	91.23	48.14	34.25	100	51.03	32.69	91.24	48.14	34.25	98	50.76	34.25	100	51.03
194	32.97	100	49.6	28.34	91.24	43.25	32.97	100	49.6	32.97	96.68	49.18	32.97	100	49.6	36.97	100	53.99
199	32.97	100	49.6	31.84	91.24	47.21	32.97	100	49.6	32.97	100	49.6	32.97	97	49.22	36.97	100	53.99
207	35.67	100	52.59	35.67	100	52.59	35.67	100	52.59	35.67	100	52.59	35.67	100	52.59	35.67	100	52.59
Avg	37.82	87.75	51.13	34.65	80.97	46.8	39.3	89.53	52.55	38.27	87.78	51.56	42.19	94.77	56.25	42.82	95.51	57.14

**Table 5:** Results of the proposed method against existing techniques on Hepatitis dataset.

t	LOF			DBSCAN			H-DBSCAN			CBLOF			LDCOF			Proposed EODA		
**P(t)**	**R(t)**	**F1(t)**	**P(t)**	**R(t)**	**F1(t)**	**P(t)**	**R(t)**	**F1(t)**	**P(t)**	**R(t)**	**F1(t)**	**P(t)**	**R(t)**	**F1(t)**	**P(t)**	**R(t)**	**F1(t)**
1	100	15.16	26.33	100	15.16	26.33	100	15.16	26.33	100	15.16	26.33	100	15.16	26.33	100	23.56	38.14
3	71.36	22.22	33.89	71.36	22.22	33.89	100	35.76	52.69	100	35.76	52.69	100	35.76	52.69	100	42.87	60.02
5	79.34	45.94	58.19	65	35.76	46.14	100	57.78	73.25	79.34	45.94	58.19	100	57.78	73.25	100	57.78	73.25
7	74.43	57.78	65.06	74.43	57.78	65.06	100	74.65	85.49	74.43	57.78	65.06	85.74	71.56	78.02	85.74	71.56	78.02
9	71.36	71.56	71.46	81.25	74.65	77.82	81.25	74.65	77.82	81.25	74.65	77.82	81.25	74.65	77.82	81.25	74.65	77.82
11	71.36	91.24	80.09	71.36	91.24	80.09	71.36	91.24	80.09	78.43	100	87.92	71.36	91.24	80.09	71.36	91.24	80.09
13	73.28	100	84.58	61.57	91.24	73.53	73.28	100	84.58	73.28	100	84.58	73.28	100	84.58	73.28	100	84.58
15	65	100	78.79	65	100	78.79	65	100	78.79	65	100	78.79	65	100	78.79	74.53	100	85.41
Avg	75.77	62.99	62.3	73.75	61.01	60.21	86.37	68.66	69.88	81.47	66.17	66.43	84.58	68.27	68.95	85.77	70.21	72.17

**Table 6:** Results of the proposed method against existing techniques on Cred dataset.

t	LOF			DBSCAN			H-DBSCAN			CBLOF			LDCOF			Proposed EODA		
**P(t)**	**R(t)**	**F1(t)**	**P(t)**	**R(t)**	**F1(t)**	**P(t)**	**R(t)**	**F1(t)**	**P(t)**	**R(t)**	**F1(t)**	**P(t)**	**R(t)**	**F1(t)**	**P(t)**	**R(t)**	**F1(t)**
11	100	24.54	39.41	100	24.54	39.41	100	24.54	39.41	100	24.54	39.41	100	24.54	39.41	100	37.56	54.61
25	86.67	61.9	72.23	79.34	55.56	65.36	84.25	61.45	71.07	95.45	69.05	80.14	95.45	69.05	80.14	95.45	69.05	80.14
55	61.4	84.78	71.23	59.65	78.56	67.82	63.68	87.63	73.76	74.93	96.34	84.3	73.68	100	84.85	74.93	96.34	84.3
63	65.34	84.78	73.81	60.77	78.56	68.53	63.79	87.63	73.84	70.69	96.34	81.55	75.45	100	86.01	75.45	100	86.01
74	51.39	87.63	64.79	52.78	90.48	66.67	57.33	91.37	70.46	58.33	100	73.69	58.33	100	73.69	58.33	100	73.69
85	47.17	87.63	61.33	47.56	91.37	62.56	47.56	91.37	62.56	54.28	100	70.37	54.28	97	69.61	54.28	99	70.12
115	36.04	95.24	52.3	36.04	95.24	52.3	44.35	100	61.45	44.35	96	60.68	44.35	97	60.87	44.35	100	61.45
245	17.43	100	29.69	17.01	96.34	28.92	17.43	100	29.69	17.43	100	29.69	17.43	100	29.69	39.12	42.25	40.63
281	23.92	100	38.61	23.92	100	38.61	23.92	100	38.61	23.92	100	38.61	23.92	100	38.61	28.67	37.56	32.52
Avg	54.38	80.73	55.94	53.01	78.97	54.47	55.82	82.67	57.88	59.94	86.92	62.05	60.33	87.51	62.55	63.4	75.76	64.83

**Table 7:** Results of the proposed method against existing techniques on Heart dataset.

t	LOF			DBSCAN			H-DBSCAN			CBLOF			LDCOF			Proposed EODA		
**P(t)**	**R(t)**	**F1(t)**	**P(t)**	**R(t)**	**F1(t)**	**P(t)**	**R(t)**	**F1(t)**	**P(t)**	**R(t)**	**F1(t)**	**P(t)**	**R(t)**	**F1(t)**	**P(t)**	**R(t)**	**F1(t)**
7	79.34	23.78	36.6	79.34	23.78	36.6	100	31.23	47.6	79.34	23.78	36.6	100	31.23	47.6	100	31.23	47.6
11	79.34	54	64.27	90	56.35	69.31	90	56.23	69.22	90	56.35	69.31	79.34	54	64.27	100	61.78	76.38
17	57.89	68.75	62.86	52.63	61.78	56.84	78.95	92.45	85.17	78.95	92.45	85.17	78.95	92.45	85.17	78.95	92.45	85.17
22	65	75	69.65	54.83	61.78	58.1	78.43	92.45	84.87	79.34	100	88.48	79.34	100	88.48	79.34	100	88.48
29	44.12	92.45	59.74	41.45	82.35	55.15	47.06	100	64.01	47.06	100	64.01	47.06	100	64.01	47.06	96	63.16
37	45.74	100	62.77	37.14	82.35	51.2	45.74	100	62.77	45.74	97	62.17	45.74	100	62.77	45.74	100	62.77
67	25.4	100	40.52	25.4	100	40.52	25.4	100	40.52	25.4	100	40.52	25.4	100	40.52	32.04	100	48.54
Avg	56.69	73.43	56.63	54.4	66.92	52.54	66.52	81.77	64.88	63.69	81.37	63.76	65.12	82.53	64.69	69.02	83.07	67.45

**Table 8:** Results of the proposed method against existing techniques on Lymp dataset.

t	LOF			DBSCAN			H-DBSCAN			CBLOF			LDCOF			Proposed EODA		
**P(t)**	**R(t)**	**F1(t)**	**P(t)**	**R(t)**	**F1(t)**	**P(t)**	**R(t)**	**F1(t)**	**P(t)**	**R(t)**	**F1(t)**	**P(t)**	**R(t)**	**F1(t)**	**P(t)**	**R(t)**	**F1(t)**
2	100	21.45	35.33	100	21.45	35.33	100	21.45	35.33	100	21.45	35.33	100	21.45	35.33	100	21.45	35.33
4	100	54	70.13	100	54	70.13	100	54	70.13	100	54	70.13	100	54	70.13	100	54	70.13
6	79.34	71.56	75.25	79.34	71.56	75.25	79.34	71.56	75.25	79.34	71.56	75.25	79.34	71.56	75.25	100	84.78	91.77
8	57.14	71.56	63.55	74.43	84.78	79.27	74.43	84.78	79.27	74.43	84.78	79.27	74.43	84.78	79.27	74.43	84.78	79.27
10	44.44	71.56	54.83	71.36	100	83.29	71.36	100	83.29	55.56	84.78	67.13	71.36	100	83.29	71.36	100	83.29
12	36.36	71.56	48.22	57.33	100	72.88	57.33	100	72.88	57.33	100	72.88	57.33	100	72.88	57.33	100	72.88
14	47	100	63.95	47	100	63.95	47	100	63.95	47	100	63.95	47	100	63.95	47	100	63.95
Avg	66.33	65.96	58.76	75.64	75.97	68.59	75.64	75.97	68.59	73.38	73.8	66.28	75.64	75.97	68.59	78.59	77.86	70.95

**Table 9:** Results of the proposed method against existing techniques on Grem dataset.

t	LOF			DBSCAN			H-DBSCAN			CBLOF			LDCOF			Proposed EODA		
**P(t)**	**R(t)**	**F1(t)**	**P(t)**	**R(t)**	**F1(t)**	**P(t)**	**R(t)**	**F1(t)**	**P(t)**	**R(t)**	**F1(t)**	**P(t)**	**R(t)**	**F1(t)**	**P(t)**	**R(t)**	**F1(t)**
11	42.85	31.87	36.56	16.67	14.29	15.39	42.85	31.87	36.56	44.56	35.74	39.67	44.56	35.74	39.67	42.85	39.87	41.31
22	25	40.45	30.91	20.83	35.74	26.33	25	40.45	30.91	29.17	54	37.88	44.56	72.94	55.33	42.85	55.56	48.39
33	20.59	54	29.82	14.74	35.74	20.88	23.53	55.56	33.06	41.45	91.37	57.03	41.18	100	58.34	41.62	77.927	54.27
45	19.44	54	28.59	16.67	40.45	23.61	27.76	55.56	37.03	44.56	100	61.65	44.56	100	61.65	42.85	85.74	57.15
48	18.42	54	27.47	15	40.45	21.89	20	55.56	29.42	35	100	51.86	35	97	51.44	41.36	91.37	56.95
56	16.67	54	25.48	14.29	40.45	21.12	21.43	64.29	32.15	42.85	94	58.87	42.85	100	60	42.85	100	60
69	13.85	64.29	22.8	16.67	55.56	25.65	21.57	100	35.49	21.57	100	35.49	21.57	97	35.3	21.57	100	35.49
115	12.39	100	22.05	8.85	72.94	15.79	12.39	100	22.05	12.39	97	21.98	12.39	100	22.05	27.56	98	43.03
227	19.34	100	32.42	19.34	100	32.42	19.34	100	32.42	19.34	100	32.42	19.34	100	32.42	24.54	100	39.41
Avg	20.95	61.41	28.46	15.9	48.41	22.57	23.77	67.04	32.13	32.33	85.79	44.1	34.01	89.19	46.25	36.45	83.17	48.45

**Table 10:** Results of the proposed method against existing techniques on Monks dataset.

t	LOF			DBSCAN			H-DBSCAN			CBLOF			LDCOF			Proposed EODA		
**P(t)**	**R(t)**	**F1(t)**	**P(t)**	**R(t)**	**F1(t)**	**P(t)**	**R(t)**	**F1(t)**	**P(t)**	**R(t)**	**F1(t)**	**P(t)**	**R(t)**	**F1(t)**	**P(t)**	**R(t)**	**F1(t)**
2	100	25	40	100	25	40	100	25	40	100	25	40	100	25	40	100	37	54.02
7	100	46	63.02	100	46	63.02	100	46	63.02	100	46	63.02	100	46	63.02	100	46	63.02
28	87.25	82.34	84.73	68	68	68	79.34	82	80.65	100	100	100	95	96	95.5	95	96	95.5
34	83.34	90.27	86.67	74.43	82	78.04	78.43	82.34	80.34	84.43	97	90.28	84.43	100	91.56	85.74	96	90.59
38	79.31	90.27	84.44	68.97	82	74.93	75.45	82.34	78.75	84.67	97	90.42	84.67	98	90.85	84.67	100	91.7
41	74.43	100	85.35	57.14	82	67.35	67.34	86	75.54	74.43	100	85.35	74.43	99	84.98	74.43	99	84.98
87	30.93	100	47.25	30.93	100	47.25	25.81	96	40.69	30.93	100	47.25	30.93	100	47.25	37.67	100	54.73
108	32.67	100	49.26	32.67	100	49.26	32.67	100	49.26	32.67	100	49.26	32.67	100	49.26	32.67	100	49.26
Avg	73.5	79.24	67.59	66.52	73.13	60.99	69.88	74.96	63.54	75.9	83.13	70.7	75.27	83	70.31	76.28	84.25	72.98

The proposed EODA incorporates several controllable hyperparameters across its three stages, significantly influencing its performance and adaptability. In the Boruta-RF feature selection stage, the primary hyperparameters include the number of decision trees ($n_{trees}$), which controls the accuracy and stability of feature importance estimation, and the maximum number of iterations ($n_{iter}$), which determines the extent of exploration for feature relevance. Additionally, the threshold for Z-score comparison affects the sensitivity of feature selection by controlling the trade-off between retaining relevant features and removing noise. In the KNN clustering stage, key hyperparameters include K, the number of nearest neighbours, which determines the neighbourhood size and influences the clustering accuracy. Smaller values of K capture local structures but may lead to overfitting, while larger values provide smoother clustering results at the risk of underfitting. The distance metric (e.g., Euclidean or Manhattan) also plays a critical role, with the choice depending on the dataset’s characteristics, and the threshold for neighbourhood consideration ($\text{Thres}{\text{h}}_{k}$) defines the maximum number of neighbours to evaluate, balancing local density and global spread.

The density function’s parameters are key hyperparameters in the outlier detection stage. The density function aggregates instance distances within a cluster, and its sensitivity can be adjusted through scaling or normalization to highlight local or global density variations. The distance weighting in the outlier score computation, which combines density and distance, also significantly impacts the detection of anomalies, offering flexibility to emphasize either highly dense or sparse regions. Furthermore, the number of outliers ($N_{top}$) selected from the sorted outlier scores is a critical parameter, directly influencing the number of anomalies detected. These hyperparameters can be tuned using methods such as grid search or cross-validation to ensure optimal performance, allowing EODA to adapt effectively to diverse datasets and anomaly detection tasks. This detailed discussion provides clarity on the controllable aspects of the proposed method and enhances its reproducibility.

A comparative study of the proposed EODA algorithm with existing algorithms in terms of the average precision, recall, and F1-Score is discussed in Tables 11, 12, and 13, respectively. The results show that the EODA algorithm consistently outperforms other outlier detection methods. In terms of precision (Table 11), which measures how accurately the algorithm identifies true outliers, EODA comes out on top with an average of 63.07%, slightly ahead of LDCOF at 61.14%. This means EODA does a better job of minimizing false positives. When it comes to recall (Table 12), which indicates how well the algorithm catches actual outliers, EODA performs strongly with an average score of 82.49%, just shy of LDCOF’s 83.83%. While LDCOF has a slight edge here, EODA remains highly reliable. The F1 score (Table 13), which balances precision and recall, highlights EODA’s strength, achieving the highest average score of 64.53%. This shows that EODA not only accurately detects outliers but also maintains consistency across different datasets. These results highlight the proposed method’s ability to handle outliers more effectively than existing techniques. Furthermore, the EODA algorithm provides a more balanced approach for detecting outliers compared to other algorithms.

**Table 11:** Comparison of the EODA algorithm with existing algorithms in terms of precision.

Algorithms	Precision (%)								
i	ii	iii	iv	v	vi	vii	viii	Avg
LOF [5]	40.40	37.82	75.77	54.38	56.69	66.33	20.95	73.50	53.23
DBSCAN [22]	39.70	34.65	73.75	53.01	54.4	75.64	15.90	66.52	51.70
H-DBSCAN [13]	47.10	39.30	86.37	55.82	66.52	75.64	23.77	69.88	58.05
CBLOF [11]	49.80	38.27	81.47	59.94	63.69	73.38	32.33	75.90	59.35
LDCOF [12]	52.00	42.19	84.58	60.33	65.12	75.64	34.01	75.27	61.14
Proposed EODA	52.30	42.82	85.77	63.40	69.02	78.59	36.45	76.28	63.07

**Table 12:** Comparison of the EODA algorithm with existing algorithms in terms of recall.

Algorithms	Recall (%)								
i	ii	iii	iv	v	vi	vii	viii	Avg
LOF [5]	72.60	87.75	62.99	80.73	73.43	65.96	61.41	79.24	73.01
DBSCAN [22]	71.60	80.97	61.01	78.97	66.92	75.97	48.41	73.13	69.62
H-DBSCAN [13]	81.20	89.53	68.66	82.67	81.77	75.97	67.04	74.96	77.72
CBLOF [11]	87.00	87.78	66.17	86.92	81.37	73.80	85.79	83.13	81.49
LDCOF [12]	89.40	94.77	68.27	87.51	82.53	75.97	89.19	83.00	83.83
Proposed EODA	90.10	95.51	70.21	75.76	83.07	77.86	83.17	84.25	82.49

**Table 13:** Comparison of the EODA algorithm with existing algorithms in terms of F1-Score.

Algorithms	F1-Score (%)								
i	ii	iii	iv	v	vi	vii	viii	Avg
LOF [5]	48.50	51.13	62.30	55.94	56.63	58.76	28.46	67.59	53.66
DBSCAN [22]	48.00	46.80	60.21	54.47	52.54	68.59	22.57	60.99	51.77
H-DBSCAN [13]	55.40	52.55	69.88	57.88	64.88	68.59	32.13	63.54	58.11
CBLOF [11]	59.70	51.56	66.43	62.05	63.76	66.28	44.10	70.70	60.57
LDCOF [12]	61.90	56.25	68.95	62.55	64.69	68.59	46.25	70.31	62.44
Proposed EODA	62.30	57.14	72.17	64.83	67.45	70.95	48.45	72.98	64.53

**Fig 3:**
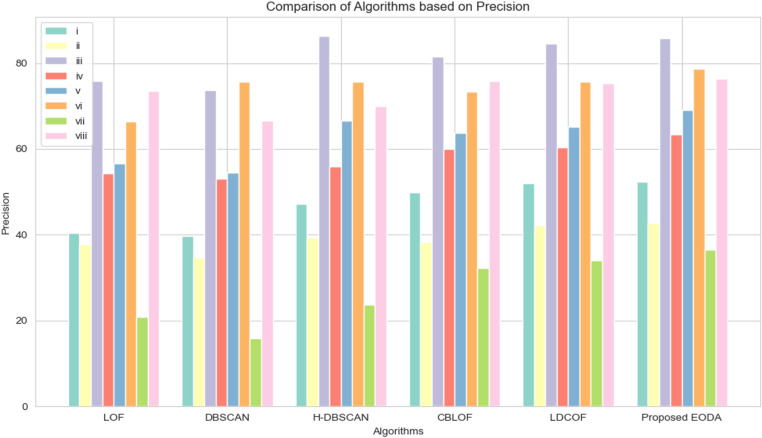
Analysis of the proposed method with the other state of the art methods based on precision.

**Fig 4:**
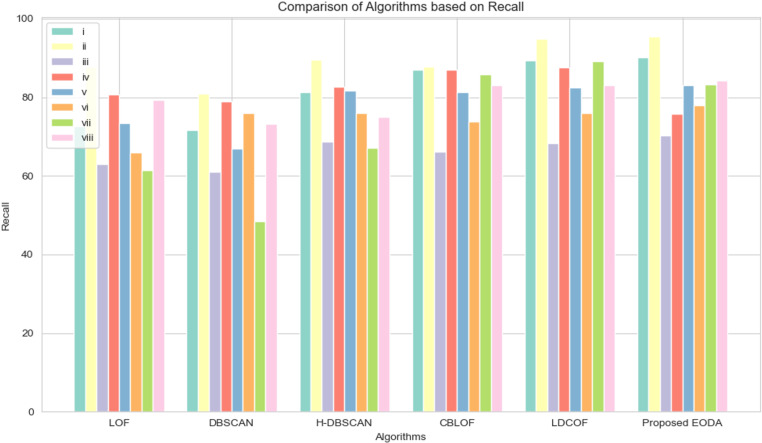
Analysis of the proposed method with the other state-of-the-art methods based on recall.

**Fig 5:**
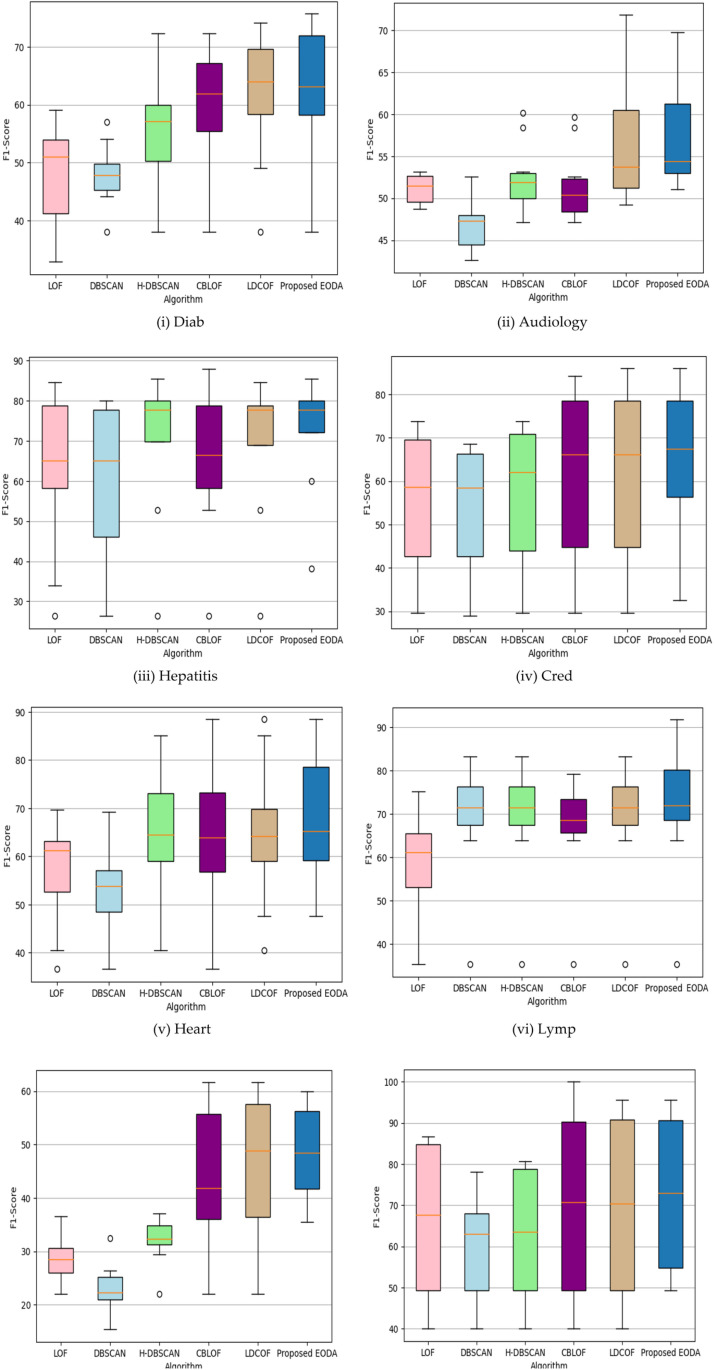
Rectangular and Notched boxplots of different detection methods over eight UCI datasets.

To evaluate the effectiveness of different outlier detection techniques, we conducted a thorough analysis using eight diverse UCI datasets. The comparison focused on key performance metrics (precision, recall, and F1-Score) presented through bar charts and boxplots to visualize the performance distribution across the algorithms. Figs 3 and 4 highlight the comparative performance of the proposed EODA method against other methods. Fig 3 focuses on precision, demonstrating how EODA consistently surpasses existing methods, showing higher precision rates across all datasets. This indicates that EODA is more effective at minimizing false positives and accurately identifying true outliers. Fig 4 presents the recall rates, which measure how effectively the methods catch actual outliers. In Fig 5, rectangular and notched boxplots provide a more detailed data-specific comparison to visualize the distribution of performance metrics across each technique and each dataset. The notched boxplots reveal that EODA not only performs better on average but also maintains less variability in its performance, indicating robustness and stability across different types of datasets. The analysis shows that EODA provides a more balanced and effective approach to outlier detection.

## 5. Conclusions

In this paper, a three-sage efficient outlier detection approach, named EODA, has been presented. The proposed EODA algorithm follows a structured three-stage process to address the challenges of the existing techniques. In the first stage, the Boruta method combined with a random forest ensemble classifier is used for feature selection, to extract all relevant features from the dataset. This ensures that only the most important attributes are considered for subsequent analysis, reducing noise and improving the quality of the data. In the second stage, the selected features are clustered using the KNN algorithm, which groups the data based on similarities. Finally, the third stage introduces an efficient outlier detection mechanism that identifies the most significant outliers within the clustered data. This stage also computes the degree of outliers for each data point, ensuring a precise and accurate identification of anomalies.

The effectiveness of the proposed EODA algorithm has been evaluated on eight UCI datasets through several key performance metrics including precision, recall, detection rate, accuracy, and F1-Score. These metrics demonstrate the method’s ability to not only detect outliers but also to do so with a high level of accuracy and reliability across different datasets. Compared to existing methods, EODA shows promising results, making it a robust tool for outlier detection in various domains.

Future research can concentrate on feature selection and clustering strategies to improve the efficiency and scalability of outlier detection. This may entail creating complicated feature selection algorithms that take a variety of data types into account and incorporate domain-specific information. Anomaly detection accuracy can also be greatly increased by analyzing advanced clustering techniques that may identify complicated data structures and take noise and outliers into consideration. The progress of outlier detection approaches made possible by these research initiatives will allow for the more robust and accurate detection of anomalies across a range of application domains. As another future work, we will prioritize addressing the current limitations by expanding our evaluation to include real-world and high-dimensional datasets, ensuring broader validation and practical applicability of the proposed approach.
